# Orbital decompression improves visual function and macular blood perfusion status in patients with thyroid-related eye disease

**DOI:** 10.3389/fmed.2024.1455226

**Published:** 2024-10-28

**Authors:** Bingxuan Wu, Min Zhu, Feng Zhang, Jiamin Cao, Bingyu Xie, Ziyi Zhu, Wei Xiong

**Affiliations:** Department of Ophthalmology, The Third Xiangya Hospital, Central South University, Changsha, China

**Keywords:** thyroid-related eye disease, orbital decompression surgery, OCTA, best corrected visual acuity, macular blood

## Abstract

**Background:**

Orbital decompression surgery is a treatment option for Thyroid-associated Ophthalmopathy (TAO). However, its effects on visual function and macular perfusion status remain unclear. This study examined how orbital decompression surgery affects visual acuity and macular blood flow in TAO patients and the variation between these two factors.

**Methods:**

This study involved cross-sectional and longitudinal analyses of 54 TAO patients, who were categorized into a normal vision group (*n* = 40) and an impaired vision group (*n* = 14) based on the presence of visual impairment before surgery (LogMAR BCVA >0.097). Among the participants, 20 underwent orbital decompression surgery (normal vision group: *n* = 13, impaired vision group: *n* = 7). BCVA and IOP were assessed at baseline in patients with TAO, and macular retinal blood perfusion indices were measured using OCTA. One month post-operatively, changes in these indices were compared between the two groups, and the correlation between visual acuity and macular perfusion indices was analyzed.

**Results:**

The LogMar BCVA in the visual impairment group was significantly worse than that in the normal vision group (*p* < 0.001), while the W-MVD was significantly lower (*p* = 0.01). In the visual impairment group, post-operative LogMar BCVA improved significantly (*p* = 0.038), and W-MVD increased significantly (*p* = 0.008) compared to baseline. However, there were no significant changes in LogMar BCVA, S-MVD, D-MVD, or W-MVD in the normal vision group from preoperative to postoperative assessments. A strong negative correlation was found between the changes in W-MVD and changes in LogMar BCVA in the eyes of the visual impairment group treated with orbital decompression (Pearson correlation coefficient, R = −0.812, *p* = 0.05).

**Conclusion:**

This study found that TAO patients with visual impairment had lower macular blood flow perfusion than those with normal vision. Endoscopic orbital decompression surgery was found to improve both the best-corrected visual acuity and macular blood flow perfusion in TAO patients with visual impairment, and the improvement in visual acuity was correlated with the improvement in macular blood flow perfusion.

## Background

Thyroid-associated Ophthalmopathy (TAO) is a thyroid-related autoimmune disease, and its pathogenesis is unclear (1). The primary mechanism involves activating T and B lymphocytes to stimulate fibroblasts in the eye socket (2). Activated fibroblasts proliferate and differentiate, producing excess glycosaminoglycans and hyaluronic acid; the thyroid-stimulating hormone receptor (TSHR) and insulin-like growth factor-1 receptor (IGF-1R) bind to receptors to initiate an inflammatory cascade, leading to edema and fibrosis of the eye socket tissue (1). Relevant studies have shown that visual impairment caused by TAO is associated with intraorbital fat hyperplasia and extraocular muscle hypertrophy, thereby compressing the eye and causing ocular blood flow obstruction (3). The macular blood flow perfusion is a crucial indicator of the severity of TAO, and serves as an objective measure for monitoring the progression of the disease (4). The retinal vascular system is the window to the ocular blood circulation. Optical coherence tomography angiography (OCTA) is a fast, convenient, non-invasive, and straightforward test that quantitatively measures retinal blood flow by identifying blood flow signals. For example, Xiaohan Zhang clarified that the severity of TAO was negatively correlated with macular central sulcus vessel density by OCTA (4). Bei Xu confirmed using OCTA that TAO visual field is positively correlated with macular blood flow perfusion, suggesting that the state of macular blood flow perfusion may affect TAO visual function (5, 6).

26.7% of TAO patients exhibit visual field (VF) defects, while 5% demonstrate a reduction in Best Corrected Visual Acuity (BCVA) (7), TAO patients exhibit significant individual variability (8). BCVA is an index for evaluating patients’ visual function that can be affected by retinal blood perfusion. For example, the mean BCVA of patients with diabetic retinopathy shows a correlation with retinal blood perfusion status. It has been demonstrated that orbital decompression significantly improves BCVA in patients with TAO (9); The effect of orbital decompression on macular blood perfusion in TAO patients and the link between postoperative visual acuity and macular blood perfusion remain unclear. Future research should aim to enhance the efficacy of orbital decompression surgery, personalize treatment approaches, and utilize innovative technologies to improve patient outcomes and quality of life. However, the correlation in visual function and retinal blood perfusion in TAO patients after orbital decompression is unclear (1).

This study aimed to explore the effects of orbital decompression surgery on best-corrected visual acuity and macular haemoperfusion in patients with TAO and to clarify the correlation between these two indices.

## Research objectives and methods

### Inclusion and exclusion criteria

Patients diagnosed with TAO at the Department of Ophthalmology, The Third Xiangya Hospital, were recruited for this study between March 2023 and March 2024. The criteria for inclusion and exclusion are presented in Table 1.

**Table 1:** Inclusion and exclusion criteria.

Serial number	Inclusion
1	According with Bartly’s diagnostic criteria (8)
2	No hormonal therapy, immunosuppressants, orbital radiotherapy within three months
3	5 tests of thyroid function were normal in the last three months after medical or surgical treatment

	Exclusion criteria
1	Presence of circulatory and metabolic disorders^2^
2	Ocular conditions other than TAO that may influence retinal blood flow or visual function
3	Smoking and alcohol abuse^3^
4	Long-term use of medications that could affect retinal blood flow or thyroid hormone levels
5	Pregnancy or breastfeeding status

Exegesis: 1: 5 tests of thyroid function include Triiodothyronine (T3), thyroxine (T4), Thyroid-stimulating hormone (TSH), Free Triiodothyronine (FT3), free thyroxine (FT4). 2: Presence of circulatory and metabolic disorders include diabetes, hypertension, and cardiovascular disease. 3: There was smoking and heavy drinking within 3 months.

Demographic data, BCVA, intraocular pressure (IOP), superficial macular blood flow density (S-MVD), deep macular blood flow density (D-MVD), and whole-layer macular blood flow density (W-MVD) and were collected from participants. Patients were categorized into a normal vision group (LogMar BCVA ≤0.097) and a visually impaired group (LogMar BCVA >0.097) based on the presence or absence of preoperative visual impairment. Participants were followed for 1 month postoperatively. This study was approved by the Scientific Ethics Committee of the Third Xiangya Hospital of Central South University, and informed consent was obtained from all participants.

### Orbital decompression surgery

The commonly employed surgical techniques for patients with TAO include Medial wall decompression alone; balanced decompression of both the medial and lateral walls; lateral wall decompression alone. The surgical indications outlined in the 2021 EUGOGO guidelines (10) were used to determine the appropriateness of orbital decompression surgery for the patients. These indications included:

1. The exophthalmos in both eyes notably affected appearance (with a side-to-side difference greater than 2 mm and bilateral measurements exceeding 14 mm), along with sensations of foreign body presence and increased peribulbar tension (11).
2. Eyeball protrusion resulting in exposure keratitis.
3. Compressive optic neuropathy, visual field defects, or vision loss attributed to hypertrophy of the extraocular muscles compressing the optic nerve.

Medial orbital wall decompression was performed for TAO patients exhibiting eye protrusion of less than 18 mm. Nevertheless, combined double-wall decompression (medial and lateral orbital walls) was indicated for those with eye protrusion exceeding 18 mm (12, 13). The operative eye is the one with a more prominent eyeball or significantly impaired visual function. All surgical procedures were conducted by the same chief oculoplastic specialist experienced in orbital diseases.

Lateral Orbital Wall Decompression Procedure (14): A horizontal skin incision was made from the lateral side of the affected eye, extending outward. The subcutaneous tissues were carefully separated until the periosteum of the orbital bone was reached. The periosteum was incised, and electrocoagulation was used to peel it away from the temporalis muscle to control hemorrhage, thereby isolating and exposing the lateral orbital wall. A reciprocating saw under a microdynamic system was used to resect approximately 2.5–3 cm of the lateral orbital rim bone tissue. The bone was thinned for potential future use. The procedure continued with the removal of bone from the deeper portions adjacent to the pterygopalatine fossa, allowing the exposure of the orbital fat within the superior and inferior muscle cones. A portion of the orbital fat was excised following adequate hemostasis. The bone defect at the lateral orbital rim was repaired using a titanium plate and two titanium screws, restoring the continuity of the lateral orbital bone with one titanium nail and the original lateral orbital bone. Closure was performed in layers, including the periosteum, subcutaneous tissues, and skin, using 5–0 absorbable sutures, and rubber drainage strips were placed.

Medial Orbital Wall Decompression Procedure: The procedure commenced with the preparation of cotton pads in a solution of 3 ml epinephrine and 6 ml lidocaine, which were introduced into the nasal cavity to induce vasoconstriction of the turbinates. An endoscopic approach was used to incise and excise the Processus uncinatus ossis ethmoidei, thereby exposing the ethmoid sinus. Access was gained to both the anterior and posterior groups of the ethmoid sinus, as well as the maxillary and sphenoidal sinuses. Strippers are adept at stripping the Lamina orbitalis labyrinthi ethmoidei (15). Fasciae of the orbit was subsequently opened; visible herniated fat was observed protruding into the ethmoid sinus. The intramuscular adipose tissue was meticulously excised, and a gelatin sponge was utilized to achieve hemostasis (Supplementary Video S1).

### Data measurement

All patients underwent assessments of BCVA, IOP, and OCTA both preoperatively and at 1-month postoperative follow-up. In non-operative patients, the eye exhibiting significant prominence was designated as the affected eye, while in operative patients, the eye receiving surgical treatment was defined as the affected eye. The OCTA examination was conducted using the Spectralis HRAPOCT2 (Heidelberg Engineering GmbH, No. 230382-004 INT.ZH20), with image processing carried out via SPECTRALISR software (version 6.12). The macular region was scanned over an area of 3 mm × 3 mm, focusing on the superficial capillary plexus (SCP) located 17 μm above the retinal nerve fiber layer (RNFL) to the inner plexiform layer (IPL), the deep capillary plexus (DCP) situated 22 μm below the IPL to the outer plexiform layer (OPL), and the full capillary plexus (FCP) extending from the internal limiting membrane (ILM) to the OPL. Capillary density (VD) was defined as the ratio of the retinal microvascular perfusion area within the measurement area (3 mm × 3 mm) to the total measurement area. The device automatically eliminates projection artifacts and calculates vessel density in deeper layers while adjusting the grayscale threshold for optimal image precision. The desired retinal layers were selected, and blood flow within the macular 3 mm × 3 mm area for each layer was labeled. The labeled area was then used to compute the blood flow density for each layer using Image J software; the ratio of the labeled area to the total area was considered the macular blood flow density for that specific layer.

### Statistical analysis

BCVA, IOP, S-MVD, D-MVD, and W-MVD were reported as means ± standard deviations. The Wilcoxon signed-rank test was employed to compare BCVA, IOP, and macular blood flow density across different layers among the groups of patients with TAO. A paired samples *t*-test was used to evaluate BCVA, IOP, and macular perfusion changes within each layer for both patient groups before and after surgery. Spearman’s correlation analysis was conducted to assess the relationship between the changes in macular perfusion values for each layer post-orbital decompression and the corresponding changes in BCVA. A *p*-value of less than 0.05 was considered statistically significant. Statistical analyses were performed using SPSS version 21.0.

## Results

### Demographic characteristics

Fifty-four patients (54 eyes) with TAO participated in this study, comprising 38 female patients (22 right eyes, 16 left eyes) and 16 male patients (8 right eyes, 8 left eyes). The average age of the participants was 44.0 ± 12.6 years, ranging from 22 to 75 years. Based on the presence or absence of preoperative visual impairment, the patients were categorized into a visually impaired group (*n* = 14) and a visually normal group (*n* = 40). The primary characteristics of both groups regarding TAO are summarized in Table 2. There was a significant age difference between the visually normal group and the visually impaired group (*p* < 0.001). However, no significant differences were observed in terms of gender, course of TAO or IOP (*p* = 0.061, ***p* = 0.057**, and *p* = 0.358, respectively). A substantial reduction in LogMAR BCVA was noted (*p* < 0.001), alongside a significant increase in S-MVD, D-MVD, and W-MVD in the eyes of patients in the visually normal group compared to those in the visually impaired group (*p* = 0.002, *p* < 0.001, and *p* < 0.001, respectively).

**Table 2:** Demographic and clinical characteristics of 54 TAO patients.

	Visual normal *N* = 40	Visual impairment *N* = 14	*Z*	*p*
Age (mean ± SD, years)	40.00 (26.25, 48.50)	56.00 (50.75, 57.50)	−4.297	<0.001*
Sex				
Male/Female	12/28	6/8	–	0.380
Course of TAO (mean ± SD, months)	6.00 (6.00, 24.00)	6.00 (2.75, 21.00)	−1.901	0.057
Eye category				
Left/Right	18/22	5/9	–	0.545
IOP (mmHg)	18.00 (16.00, 20.00)	20.00 (18.00, 24.25)	−1.870	0.061
BCVA (LogMar)	0.00 (−0.08, 0.00)	0.52 (0.35, 0.82)	−5.666	<0.001*
S-MVD	0.15 (0.12, 0.16)	0.11 (0.10, 0.13)	−3.117	0.002*
D-MVD	0.27 (0.23, 0.28)	0.21 (0.18, 0.23)	−3.452	<0.001*
W-MVD	0.20 (0.17, 0.23)	0.16 (0.12, 0.16)	−3.889	<0.001*

Exegesis: IOP, intraocular pressure; BCVA, best-corrected visual acuity; S-MVD, superficial macular vascular density; D-MVD, deep macular vascular density; W-MVD, whole macular vascular density. * *p* < 0.05.

### Differences between groups in preoperative comparisons

**Figure 1:**
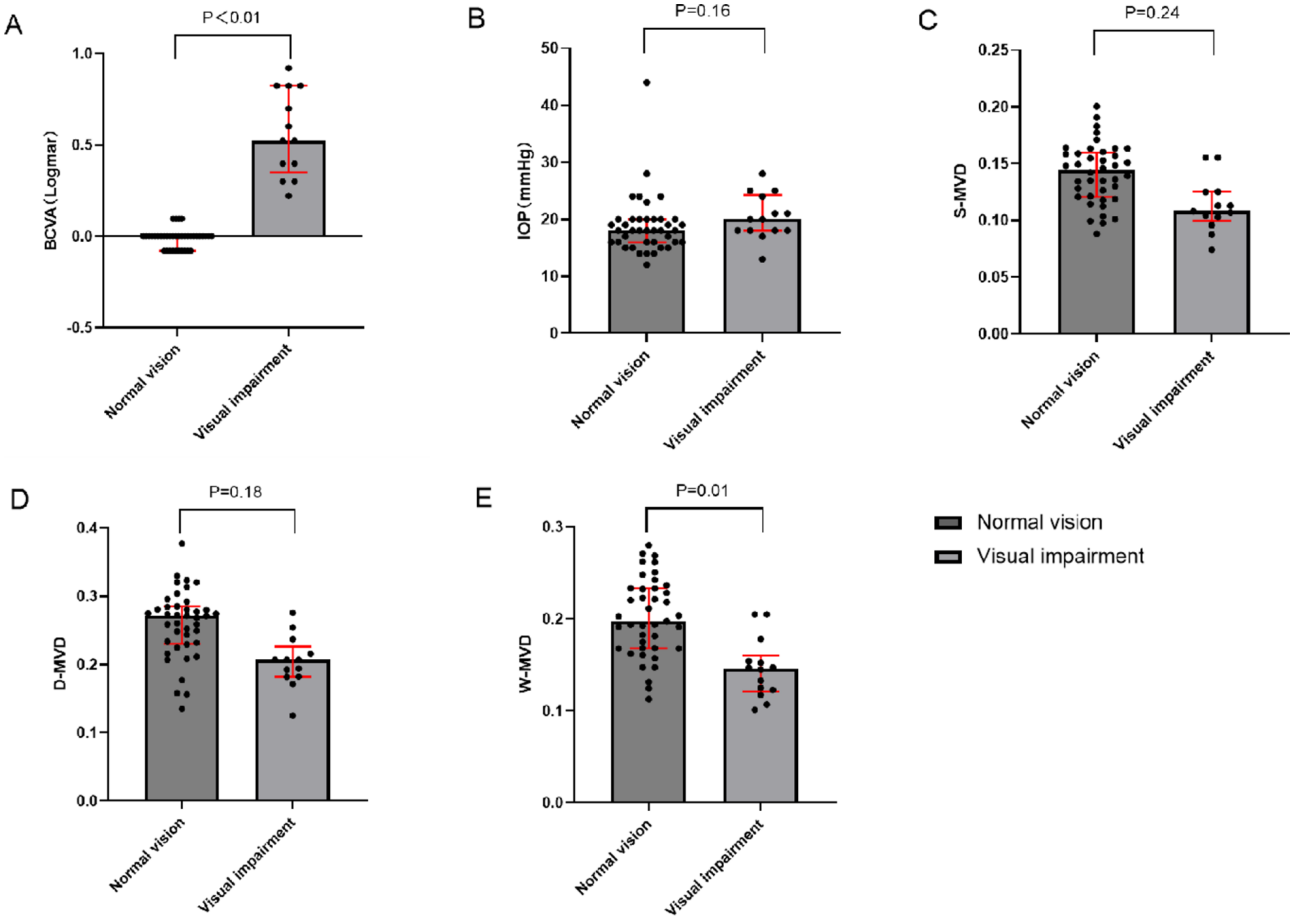
Comparison of baseline indices between impaired vision and normal vision groups of TAO patients who underwent orbital decompression. **(A)** Inter-group comparison in BCVA **(B)** Inter-group comparison in IOP **(C)** Inter-group comparison in S-MVD **(D)** Inter-group comparison in D-MVD **(E)** Inter-group comparison in W-MVD; BCVA, best-corrected visual acuity; IOP, intraocular pressure; S-MVD, superficial macular vascular density; D-MVD, deep macular vascular density; W-MVD, whole macular vascular density.

Twenty out of the 54 eyes were evaluated for orbital decompression, including 13 eyes from the normal vision group and 7 eyes from the visually impaired group. The baseline characteristics of both groups are illustrated in Figure 1. The IOP at baseline was 18.00 (16.00, 20.00) mmHg in the normal vision group and 20.00 (18.00, 24.25) mmHg in the visually impaired group, showing no significant difference between the two groups (*p* = 0.16). At baseline, the LogMAR BCVA was 0.00 (−0.08, 0.00) in the normal vision group and 0.52 (0.35, 0.82) in the visually impaired group, indicating a significant difference between the two groups (*p* < 0.01). There were no significant differences in S-MVD (*p* = 0.24) and D-MVD (*p* = 0.18) between the eyes of the normal vision group and those of the visually impaired group. However, W-MVD was significantly higher in the eyes of the normal vision group than those in the visually impaired group (*p* = 0.01) (Table 3).

**Table 3:** Clinical characteristics of TAO patients in the normal vision and visually impaired groups before performing orbital decompression surgery.

	Visual normal *N* = 13	Visual impairment *N* = 7	*Z* value	*p* value
Age (years)	38.00 (30.00, 47.00)	51.00 (49.00, 57.00)	−3.01	0.003
Sex				
Male/Female	9/4	2/5	–	0.081
Eye category				
Left/Right	5/8	2/5	–	0.658
IOP (mmHg)	19.00 (17.00, 20.00)	20.00 (18.00, 24.00)	−1.4	0.16
BCVA (LogMar)	0.00 (0.00, 0.00)	0.52 (0.30, 0.82)	−3.99	<0.01*
S-MVD	0.14 (0.10, 0.15)	0.11 (0.08, 0.13)	−1.18	0.24
D-MVD	0.26 (0.19, 0.29)	0.19 (0.17, 0.24)	−1.35	0.18
W-MVD	0.22 (0.15, 0.26)	0.13 (0.11, 0.15)	−3.71	0.01*

Exegesis: IOP, intraocular pressure; BCVA, best-corrected visual acuity; S-MVD, superficial macular vascular density; D-MVD, deep macular vascular density; W-MVD, whole macular vascular density. * *p* < 0.05.

### Intra-group analysis

**Figure 2:**
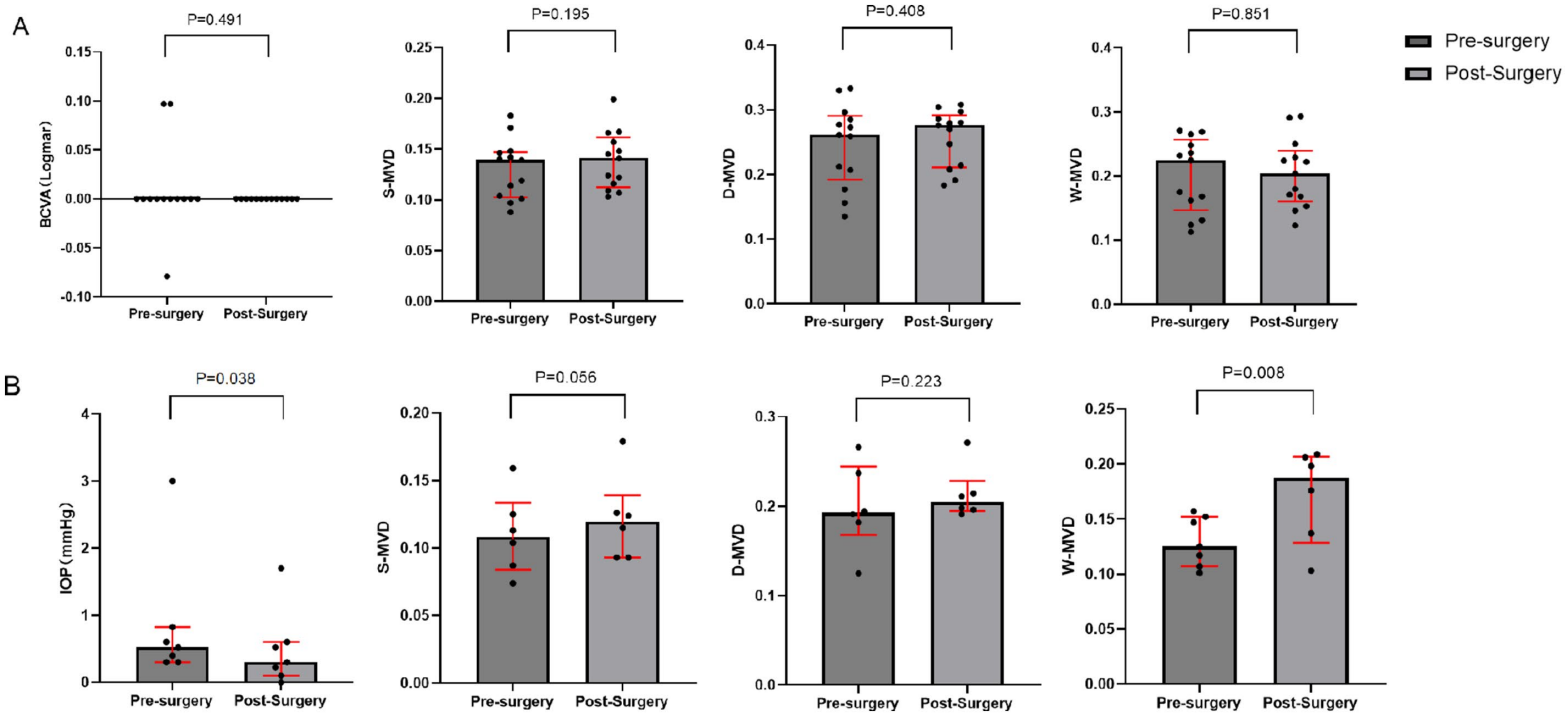
Changes in BCVA and macular retinal perfusion indices in the visually impaired and normal vision groups after orbital decompression surgery. **(A)** normal vision group; **(B)** visually impaired group.

There were no significant changes in LogMAR BCVA, S-MVD, D-MVD, and W-MVD in the normal vision group following orbital decompression surgery when compared to baseline measurements (*p* = 0.491, *p* = 0.195, *p* = 0.408, and *p* = 0.851, respectively). In the visually impaired group, the LogMAR BCVA was 0.52 (0.30, 0.82) at baseline and improved to 0.30 (0.10, 0.60) in the affected eyes 1 month postoperatively, reflecting a significant reduction from baseline (*p* = 0.038). The W-MVD of the eyes in the visually impaired group was 0.16 (0.12, 0.16) at baseline, and after 1 month of orbital decompression therapy, it increased to 0.19 (0.13, 0.21), representing a significant improvement from baseline (*p* = 0.008). Conversely, there were no significant changes in S-MVD and D-MVD compared to baseline measurements (*p* = 0.056 and *p* = 0.223, respectively) (Figure 2; Table 4).

**Table 4:** Changes in BCVA and macular retinal perfusion indices in the visually impaired and normal vision groups after orbital decompression surgery.

	Visual normal *N* = 13			Visual impairment *N* = 7		
	M (P25, P75)	*t*	*p*	M (P25, P75)	*t*	*p*
**BCVA (LogMAR)**						
Pre-op	0.00 (0.00, 0.00)	0.71	0.491	0.52 (0.30, 0.82)	2.55	0.038*
Post-op	0.00 (0.00, 0.00)			0.30 (0.10, 0.60)		
**S-MVD**						
Pre-op	0.14 (0.10, 0.15)	1.37	0.195	0.11 (0.08, 0.13)	2.36	0.056
Post-op	0.14 (0.11, 0.16)			0.12 (0.10, 0.14)		
**D-MVD**						
Pre-op	0.26 (0.19, 0.29)	0.86	0.408	0.19 (0.17, 0.24)	1.36	0.223
Post-op	0.28 (0.21, 0.29)			0.19 (0.20, 0.23)		
**W-MVD**						
Pre-op	0.22 (0.15, 0.26)	0.19	0.851	0.16 (0.12, 0.16)	3.94	0.008*
Post-op	0.20 (0.16, 0.24)			0.19 (0.13, 0.21)		

Exegesis: Pre-op, pre-operative; Post-op, postoperative; BCVA, best-corrected visual acuity; S-MVD, superficial macular vascular density; D-MVD, deep macular vascular density; W-MVD, whole macular vascular density; *, *p* < 0.05.

### Correlation of BCVA with macular blood flow

**Figure 3:**
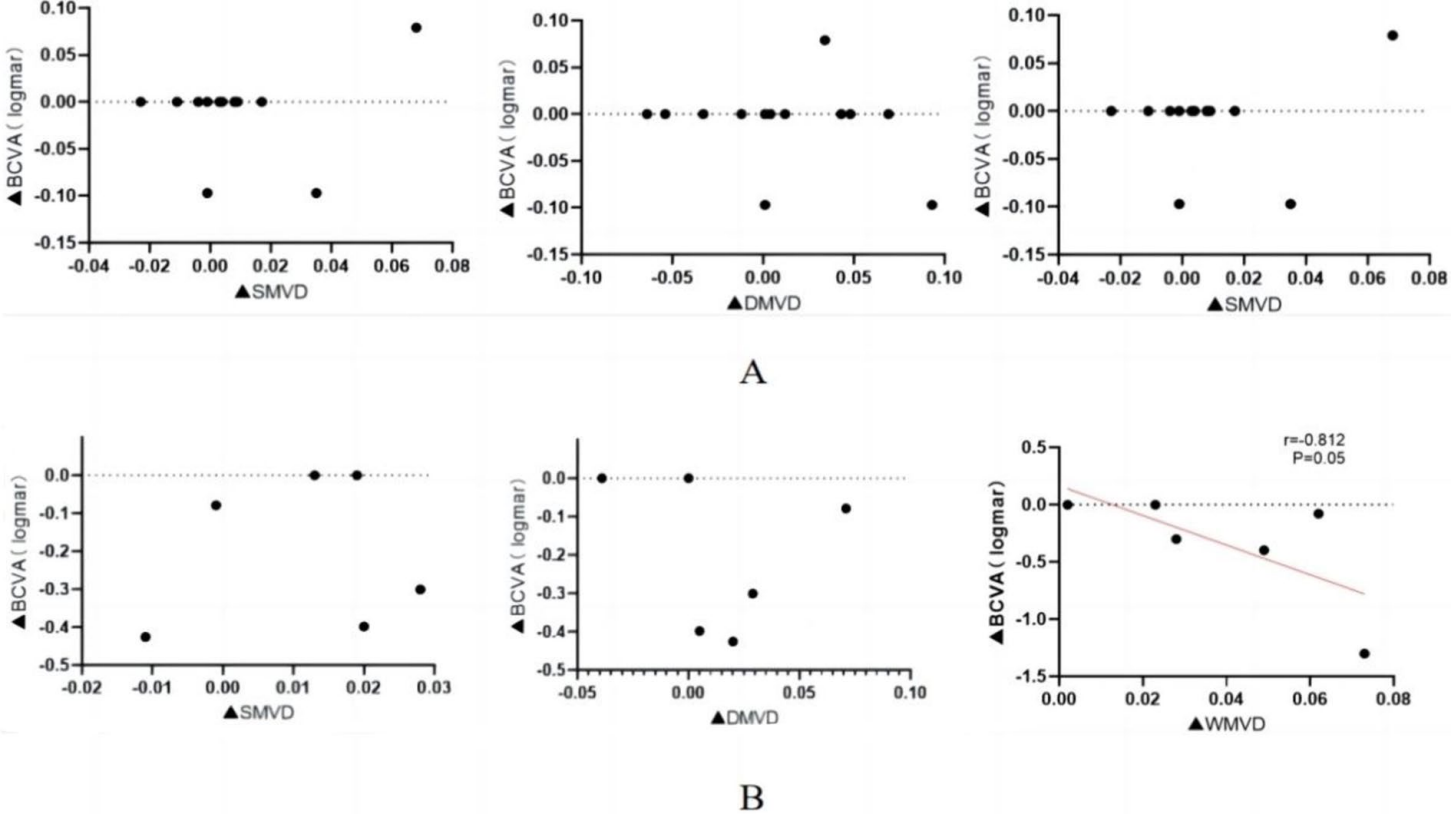
Correlation of macular perfusion change values with LogMar BCVA change values after orbital decompression surgery treatment in the visually impaired group. **(A)** normal vision group; **(B)** visually impaired group. ▲: postoperative-baseline.

A Spearman’s correlation analysis was performed to assess the relationship between changes in LogMAR BCVA and alterations in macular retinal perfusion indices across both study groups. The findings are summarized in Table 5 and illustrated in Figure 3. In the normal vision group, no significant correlations were found between changes in LogMAR BCVA and changes in S-MVD (*p* = 0.057), D-MVD (*p* = 0.170), or W-MVD (*p* = 0.083) following orbital decompression therapy. Similarly, in the visually impaired group, no significant correlations were identified between changes in LogMAR BCVA and changes in S-MVD (*p* = 0.827) or D-MVD (*p* = 0.354) post-orbital decompression surgery. However, a strong negative correlation was observed between the change in LogMAR BCVA and the change in W-MVD in the visually impaired group (R = −0.812, *p* = 0.05), suggesting that improvements in overall macular perfusion were positively associated with enhancements in visual acuity.

**Table 5:** Spearman’s correlation analysis of the changes in retinal perfusion indices with the values of changes in BCVA after orbital decompression in both groups.

Normal visual group (*N* = 13)	*r* value	*p* value
▲BCVA (LogMAR) ——▲S-MVD	−0.539	0.057
▲BCVA (LogMAR) ——▲D-MVD	−0.405	0.170
▲BCVA (LogMAR) ——▲W-MVD	−0.498	0.083

Visual department group (*N* = 8)		
▲BCVA (LogMAR) ——▲S-MVD	0.116	0.827
▲BCVA (LogMAR) ——▲D-MVD	−0.464	0.354
▲BCVA (LogMAR) ——▲W-MVD	−0.812	0.05*

Exegesis: ▲ Difference from baseline for each indicator after surgery. *, *p* < 0.05; ——, relevance.

### Comparison of pre-and post-operative fundus photography with OCTA

**Figure 4:**
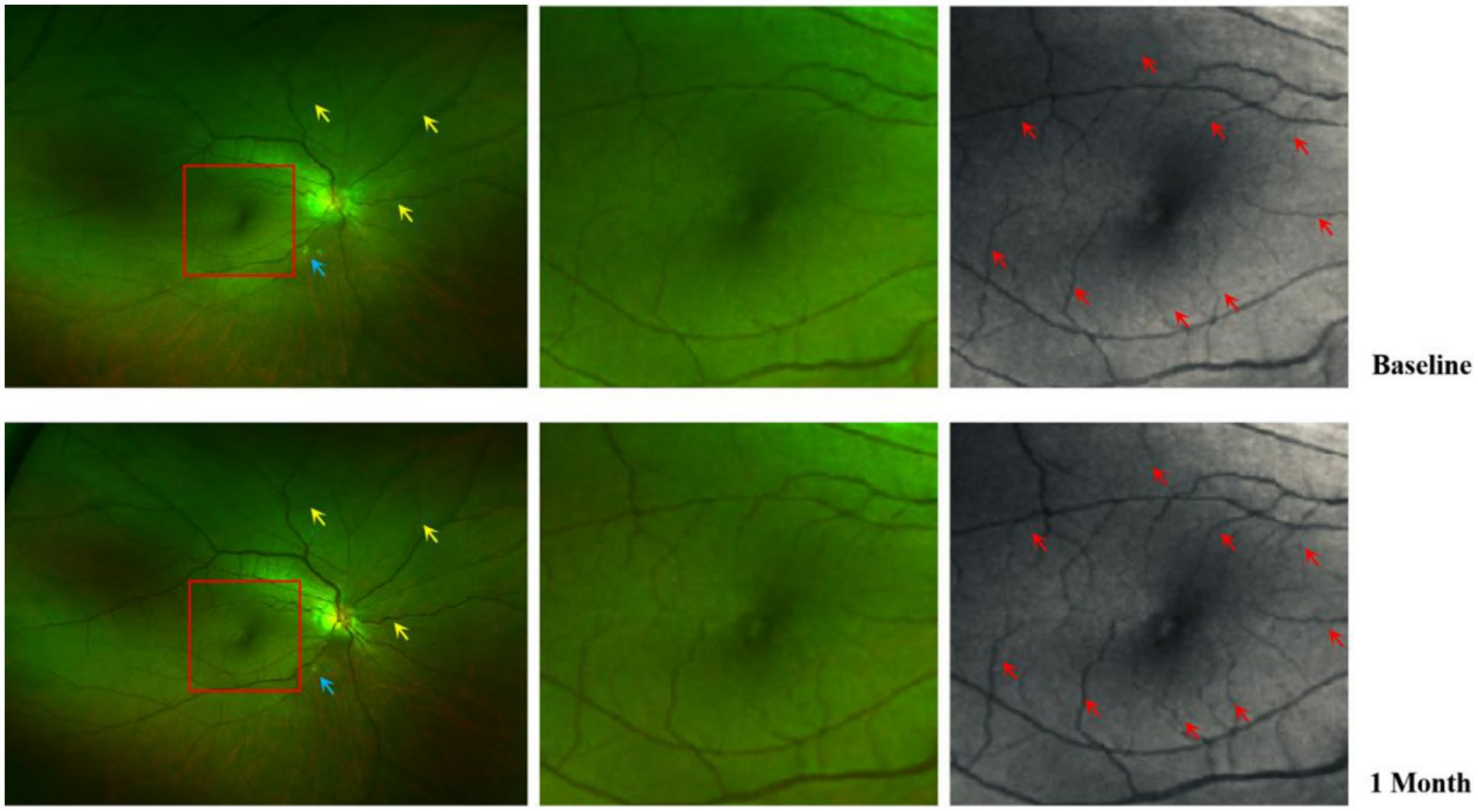
Comparison of fundus photographs of a 57-year-old female eye at baseline and after orbital decompression surgery. Yellow arrows show thickening of the caliber of some of the retinal vessels after surgery compared with before surgery; Blue arrows indicate that there are fewer cotton-padded spots after surgery than before, suggesting that there is less microretinal obstruction and less retinal ischaemia than before; Red arrows show that postoperative retinal capillary caliber in the macular region is thicker than preoperative and microcapillary perfusion is increased compared with preoperative.

**Figure 5:**
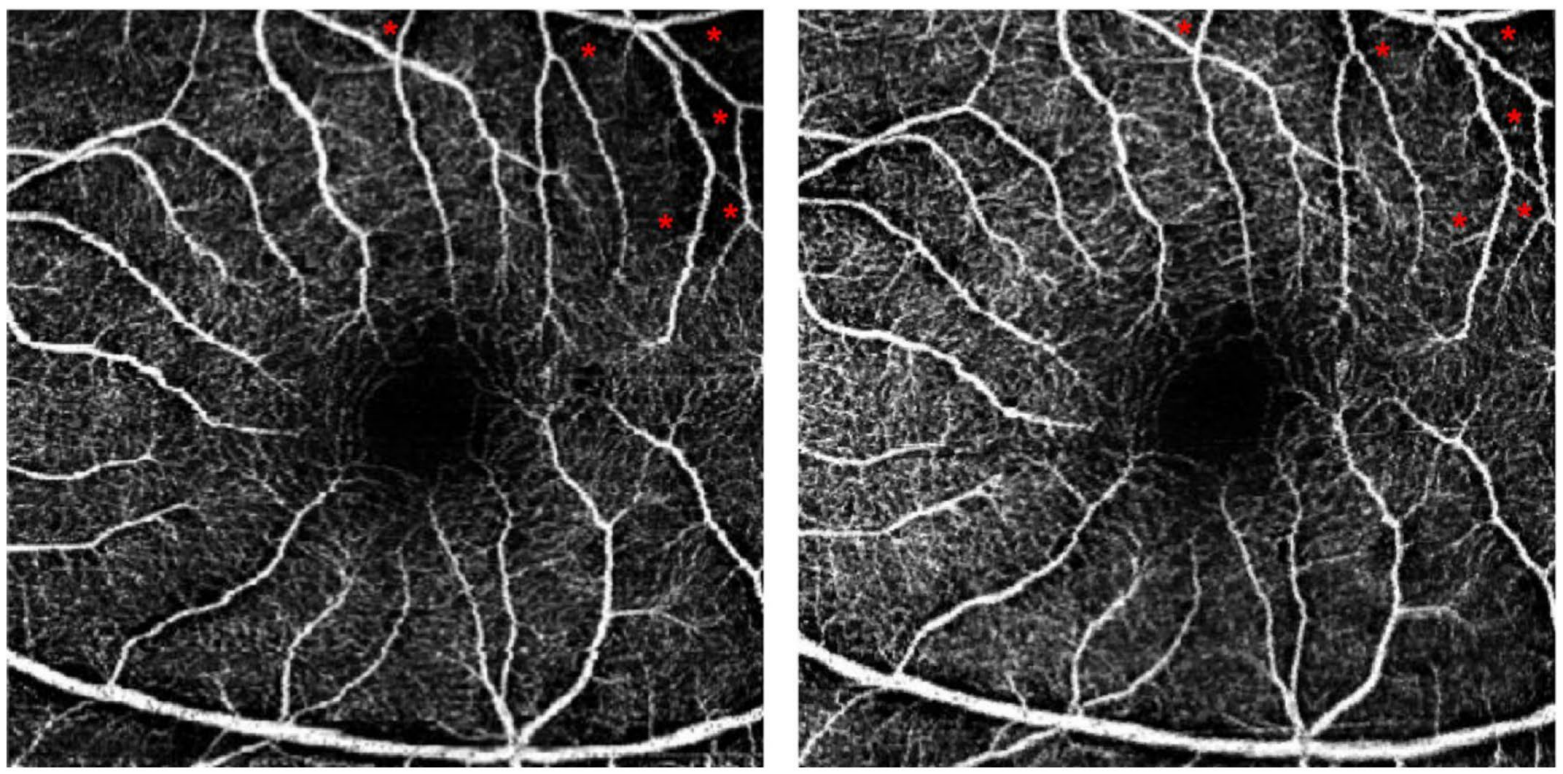
Comparison of baseline and post orbital decompression surgery treatment OCTA of a patient’s eye. **(A)** is the OCTA of the left eye of a 59-year-old male at baseline, and **(B)** is the OCTA after 1 month of treatment with orbital decompression surgery. Thickened capillaries were found at the blue arrow markings. Macular capillary caliber thickening at red markers.

Figure 4 depicts the changes in fundus photographs of a 57-year-old female patient with TAO at baseline and following orbital decompression therapy. The yellow arrow markings indicate a significant increase in the caliber of the posterior segment blood vessels after treatment. The triangular markings highlight a reduction in the area of ischemic infarction, while the rectangular area delineates a 3 × 3 mm macular region. The red arrow markings point to a notable thickening of the macular blood vessels, suggesting a potential increase in macular blood flow perfusion (Figure 4). Additionally, Figure 5 presents the optical coherence tomography angiography (OCTA) changes in a 59-year-old male patient with TAO, with blue arrow markings indicating an increase in vascular caliber in the affected eye following orbital decompression surgery.

## Discussion

In this study, it was demonstrated that whole macular perfusion was lower in TAO patients with visual impairment, and that orbital decompression therapy improved both BCVA and macular perfusion status in these patients. Furthermore, changes in visual acuity were positively correlated with alterations in whole macular perfusion. Shen Xiaojiao et al. ascertained a correlation between visual acuity and mean macular sensitivity, as well as postoperative macular thickness in treating idiopathic macular epiretinal membranes (16). Additionally, SPAIDE R F et al. demonstrated that in cases of diabetic retinopathy (DR), visual acuity in patients with diabetes mellitus improved through Optical Coherence Tomography (OCT) measurements of the Ganglion Cell Complex (GCC) (17). These findings indicate that abnormal signs in the macula are closely associated with visual acuity.

The macular perfusion status in TAO patients may indicate retinal perfusion in the posterior segment. As illustrated in Figure 4, following orbital decompression surgery in TAO patients, there was a notable enlargement of blood vessel caliber in various areas of the macula, accompanied by an increase in microcapillary perfusion compared to the preoperative period. This improvement in macular perfusion is highlighted by the red arrows. Additionally, there was a reduction in the number of cotton wool spots in the retina post-surgery, suggesting enhanced retinal perfusion in the posterior segment. Fares Antaki et al. also found that peripheral retinal perfusion indices correlated with central retinal perfusion indices, indicating that macular perfusion status may indirectly reflect the state of perfusion in the posterior retina (18).

There is a relative paucity of studies delving into the relationship between visual acuity and retinal perfusion status in patients with TAO. This study found that decreased visual acuity in TAO patients may be associated with insufficient macular perfusion. Abdelshafy M et al. demonstrated a significant positive correlation between changes in visual acuity and alterations in macular perfusion status in patients with DR (19), whereas no significant correlation was observed in the studies by Samara et al. (20) and Hwang et al. (21). The mechanism of ischemia in DR related to retinal vein occlusion (RVO) involves the upregulation and release of vascular endothelial growth factor (VEGF), which disrupts the blood-retinal barrier, leading to leakage of blood components into the tissue interstitial space and increased intravascular pressure. This results in macular edema and significantly reduced visual acuity (22). In contrast, the mechanism of ischemia in TAO patients primarily stems from hyperplasia and edema of orbital muscle and adipose tissues, which leads to increased orbital pressure and compression of intraorbital arteries and veins, ultimately resulting in decreased retinal blood flow density (4). The presence of bipolar cells and photoreceptors in the macular area means that a lack of macular perfusion can damage photoreceptors, adversely affecting visual function (23). Furthermore, when orbital pressure increases to a certain threshold, it can lead to elevated scleral venous pressure, increasing resistance to aqueous humor outflow and potentially resulting in elevated intraocular pressure (IOP). High IOP has been reported in TAO patients, with prevalence rates ranging from 3.7 to 24% (24).

Patients with TAO often present with elevated IOP, and some studies have suggested that IOP may influence the thickness of the fovea centralis in these patients (25, 26). Lee WH et al. demonstrated a correlation between macular thickness and macular perfusion in cases of hypertensive retinopathy (27), while Lommatzsch C et al. found that OCTA results in patients with glaucoma and high IOP indicated a reduced vascular density in both the optic disc and macula (28). The vascular theory of glaucoma posits that elevated IOP diminishes local ocular blood flow, particularly affecting choroidal perfusion pressure and increasing resistance to blood flow (29). However, the mean IOP in the TAO patients included in this study was within normal limits, and there were no significant changes in IOP before and after orbital decompression surgery, thereby ruling out the influence of high IOP on visual function and macular blood perfusion in these patients. Therefore, it is speculated that the primary mechanism of retinal ischemia in TAO patients is attributed to compression of the central retinal artery and the short posterior ciliary artery due to elevated intraorbital pressure. Perri et al. utilized OCTA to measure retinal blood flow in TAO patients, finding that increased retinal blood flow was associated with the volume of ocular muscles (6). Ohtsuka K. et al. reported that intraorbital lipodystrophy and increased intraorbital pressure compress the optic nerve and the central retinal artery, leading to a decrease in ocular blood supply (30). Mercé J. et al. concluded that hyperplasia of intraorbital fat, hypertrophy of extraocular muscles, progressive elevation of orbital pressure, venous reflux obstruction, and inadequate arterial blood supply collectively impair retinal blood flow perfusion density (31). The ocular blood supply originates from the ophthalmic artery, penetrating the orbit and bifurcating into two distinct vascular systems. The central retinal vascular system supplies blood to the RNFL, inner plexiform, and inner nuclear layers, while the short posterior ciliary arteries branch within the choroid to nourish the outer four layers of the retina and the optic disc, extending to the macular region through the ciliary retinal artery. Therefore, it is proposed that decreased macular perfusion in TAO patients may result from the proliferation of intraorbital adipose tissue and uneven hypertrophy of extraocular muscles, leading to elevated orbital pressure and subsequent compression of the central retinal artery and the short posterior ciliary artery. This state of retinal ischemia and hypoxia adversely affects the function of optic nerve cells, resulting in vision loss (27).

As a first-line treatment for TAO patients, orbital decompression has been shown to improve ocular protrusion, correct strabismus, adjust eyelid position, and enhance visual acuity. In this study, orbital apical decompression was incorporated with traditional nasoendoscope decompression of the medial orbital wall, which exposed the optic nerve sheath and provided additional space for the central retinal artery coursing within the optic nerve. This approach aims to further reduce pressure at the orbital apex, thereby enhancing ocular blood flow and improving retinal perfusion status.

Only a few studies have delved into the effect of orbital decompression on retinal perfusion in TAO patients. Thus, the impact of orbital decompression on retinal perfusion and visual acuity was explored in this population. Yujie Wu et al. found that superior ophthalmic veinous blood flow can stagnate in TAO patients, and that orbital decompression improves this venous flow (32). Conversely, Jia Huiwu reported a significant reduction in radial peripapillary capillary in TAO patients; although orbital decompression improved visual acuity, it did not immediately reverse the decline in radial peripapillary capillary density (33). The effect of orbital decompression on perfusion may relate to the vascular susceptibility to hypoxia, given the high metabolic demands of unmyelinated nerve fibers around the optic disc, which predispose peripheral vessels to ischemic injury compared to those in the macular region (34).

There was no significant improvement in visual acuity or macular perfusion following orbital decompression in the normal vision group compared to the visually impaired group. The degree of tissue proliferation in the normal vision group likely did not exert sufficient compressive force on the retinal blood vessels, or any mild compression may have been compensated for by increased blood flow. Consequently, no changes in retinal perfusion or visual acuity were observed postoperatively. However, when intraorbital pressure compresses blood vessels to a critical extent, this compensatory mechanism may prove inadequate in maintaining original blood flow density, resulting in retinal ischemia and hypoxia (17). Our findings indicate a correlation between changes in visual acuity following orbital decompression and changes in global macular perfusion in the visually impaired group of TAO patients, suggesting that improved whole macular perfusion may contribute to enhanced visual acuity in these eyes.

This study has notable limitations. Firstly, it is a small-sample retrospective study; further prospective and longitudinal research with larger sample sizes is required to dynamically assess changes in retinal blood flow in TAO patients following orbital decompression surgery. Secondly, all orbital decompression surgeries included in this study were performed by the same surgeon, which may introduce bias related to individual surgical techniques.

In conclusion, patients with TAO and visual impairment exhibit reduced macular perfusion status, and orbital decompression surgery may enhance visual acuity by improving the overall perfusion of macular blood flow.

## Data Availability

The original contributions presented in the study are included in the article/Supplementary material, further inquiries can be directed to the corresponding authors.
